# Anti-Inflammatory and Immunomodulatory Properties of Inorganic Fullerene-Like Tungsten Disulfide Nanoparticles in the Culture of Human Peripheral Blood Mononuclear Cells

**DOI:** 10.3390/nano15050322

**Published:** 2025-02-20

**Authors:** Snežana Zečević, Darinka Popović, Sergej Tomić, Marina Bekić, Sara Rakočević, Maja Kosanović, Dušica Stojanović, Petar Uskoković, Milan Marković, Dejan Bokonjić, Miodrag Čolić

**Affiliations:** 1 Medical Faculty Foca, University of East Sarajevo, 73300 Foča, Bosnia and Herzegovina; snezana.zecevic@ues.rs.ba (S.Z.); darinka.popovic@ues.rs.ba (D.P.); sara.rakocevic@ues.rs.ba (S.R.); dejan.bokonjic@ues.rs.ba (D.B.); 2 Institute for the Application of Nuclear Energy, University of Belgrade, 11080 Belgrade, Serbia; sergej.tomic@inep.co.rs (S.T.); marina.bekic@inep.co.rs (M.B.); maja@inep.co.rs (M.K.); milan.markovic@inep.co.rs (M.M.); 3 Faculty of Technology and Metallurgy, University of Belgrade, 11000 Belgrade, Serbia; duca@tmf.bg.ac.rs (D.S.); puskokovic@tmf.bg.ac.rs (P.U.); 4 Serbian Academy of Sciences and Arts, 11000 Belgrade, Serbia

**Keywords:** WS_2_ nanoparticles, human mononuclear cells, cytotoxicity, proliferation

## Abstract

Tungsten disulfide (WS_2_) nanoparticles have emerged in the biomedical field as potential theranostic agents due to their unique properties, including biocompatibility. However, their impact on the immune response remains unexplored. This study aimed to evaluate the effects of inorganic fullerene-like WS_2_ (IF-WS_2_) nanostructures on human peripheral blood mononuclear cells (PBMCs) in vitro. The study investigated several parameters to evaluate the effects of IF-WS_2_ nanoparticles. Cytotoxicity was assessed by measuring cell viability, apoptosis, and necrosis. Internalization of IF-WS_2_ by PBMCs was analyzed using morphological and flow cytometric techniques. Proliferation was studied in CellTrace Far Red-prestained total PBMCs stimulated with phytohemagglutinin (PHA) and in isolated T cell cultures stimulated with CD3/CD28-coated beads. Additionally, the production of cytokines and chemokines was measured in culture supernatants of total PBMCs and T cells. IF-WS_2_ nanoparticles were non-cytotoxic up to a concentration of 200 µg/mL. Concentrations ≥25 µg/mL inhibited PHA-stimulated PBMC proliferation but did not affect T cell proliferation. Morphological and flow cytometric analysis demonstrated dose- and time-dependent internalization of IF-WS_2_ by macrophages. Additionally, IF-WS_2_ significantly reduced the production of pro-inflammatory cytokines (IL-1β, TNF-α, IL-8, MCP-1, and GRO-α) in PHA-stimulated PBMCs. Th1, Th17, and Th21 cytokines were downregulated, while Th2, Th9, and T regulatory cytokines were upregulated. In conclusion, this study demonstrated for the first time that pristine IF-WS_2_ nanoparticles, at non-cytotoxic concentrations, exhibit notable anti-inflammatory and immunomodulatory properties on activated PBMCs in vitro.

## 1. Introduction

Tungsten disulfide (WS_2_) belongs to transition metal dichalcogenides [1]. Nanostructures produced from this dichalcogenide exist in the form of inorganic fullerene-like structures (IF-WS_2_) and other two-dimensional (2D) structures, such as nanotubes, nanosheets, quantum dots, different composites, or hybrids. IF-WS_2_ has quasispherical hollow polyhedral layered nanostructures similar to multiwall carbon fullerenes. They consist of two-dimensional molecular sheets assembled by van der Waals forces [2]. These basic structures give WS_2_ nanoparticles unique properties, such as lubricity, mechanical strength, and potential applications in tribology, electronics, energy storage, and other areas [3].

Due to their chemical and physical stability, WS_2_ nanoparticles are increasingly attracting biomedical research because of their special properties, such as good optical activity and conductivity. The possibility of easy functionalization or coating with different chemical and biological compounds is another advantage in enhancing their colloidal stability, dispersibility, or reactivity [4,5].

The application field of WS_2_ and other metal sulfide nanoparticles is very broad. It includes different theranostic procedures, such as magnetic resonance imaging, computed tomography, optical and photoacoustic imaging, photothermal and photodynamic therapy, and their combinations [6]. The delivery of drugs, biomolecules, or DNA to specific target tissues, particularly tumors, by 2D WS_2_ and their use as electrochemical and optical biosensors in diagnostics are dominant biomedical fields of application [4,7]. Such applications are possible and can be fine-tuned due to specific electronic band structures, luminescence, Raman scattering, and other properties of WS_2_ nanostructures [6].

The absence of toxicity is a general prerequisite for the biomedical application of nanoparticles, and in this context, WS_2_ nanostructures are considered biocompatible, as judged by the absence of cytotoxicity and genotoxicity. Such conclusions were drawn from different experiments, including rat salivary gland cells in culture [8], A549 cells [9], or human epithelial kidney cells (HEK293f) [10]. However, prolonged exposure of tumor cell lines to WS_2_ nanostructures induces dose-dependent cytotoxicity [11]. The absence of genotoxicity and genetic mutagenesis was demonstrated by evaluating the base-pair mutation in *S. typhimurium* tester strain TA100 using the Ames Fluctuation test [10].

2D transition metal dichalcogenides (2D TMDCs), in the form of nanosheets, including WS_2_, were investigated in the MH-S murine alveolar macrophage cell line culture [12]. WS_2_ nanosheets were internalized in lysosomes and triggered autophagy via the mammalian target of rapamycin (mTOR)-dependent activation pathway [12]. However, it remains to be investigated whether autophagy is a trigger of cytotoxicity or whether TMDC nanostructures may have some regulatory role in autophagy mechanisms.

The interaction of nanoparticles with the immune system is particularly important for their in vivo application. The immune system can react to foreign substances, with different effector mechanisms involved in their elimination [13]. The long persistence of nanoparticles in vivo and difficulties in their elimination can trigger a chronic immune or toxic response with many unpredictable consequences [13]. Nanoparticles can be immunotoxic or immunomodulatory, and in this context, extensive investigation is needed [13]. This is especially important when using nanoparticles in ongoing immunotherapy protocols, whose potential application has expanded tremendously due to positive results obtained in tumor therapy [6,14]. In this context, it is significant to outline that the influence of nanoparticles on the immune response is highly dependent on their size and other physicochemical properties.

Therefore, we aimed to study the effect of pristine IF-WS_2_ nanoparticles with well-defined physicochemical characteristics on peripheral blood mononuclear cells (PBMCs) in culture (cytotoxicity, nanoparticle internalization, lymphocyte proliferation, and production of pro- and anti-inflammatory cytokines, including those originating from different T cell subsets) as a first screening model of the immunomodulatory potential of nanomaterials.

## 2. Materials and Methods

### 2.1. IF-WS_2_ Nanoparticles

IF-WS_2_ nanoparticles were provided by NanoMaterials, Ltd. (ApNano Materials, Yavne, Israel). The nanoparticles were synthesized via a solid–gas reaction of metal oxide nanoparticles with H_2_/H_2_S gases at elevated temperatures (800–900 °C) using a fluidized bed reactor. Tungsten oxide powder and H_2_S gas served as reaction precursors in a reducing atmosphere of hydrogen. The detailed production process was originally described in [15].

### 2.2. Nanoparticle Tracking Analysis

To assess the size distribution of IF-WS_2_ nanoparticles, 1 mg of IF-WS_2_ was dispersed in 1 mL of either 18 mΩ double-distilled water (ddH_2_O), phosphate-buffered saline (PBS) (Sigma-Aldrich, St. Louis, MO, USA), or RPMI 1640 medium (Sigma-Aldrich) supplemented with 10% fetal calf serum (FCS) (PAA Laboratories, Vienna, Austria). Samples were sonicated for 5 min immediately before measurements with Nanoparticle Tracking Analysis (NTA, ZetaView, Particle Metrix, Inning am Ammersee, Germany).

Different dilutions and excitation lasers were pre-tested to optimize particle detection. The ideal dilution for IF-WS_2_ in ddH_2_O was 1:2000, with measurements taken at 488 nm excitation (40 mW). Each sample was analyzed by scanning 11 positions with 30 frames per position. Measurement settings included sensitivity: 85, shutter: 2000, and cell temperature: 24.9 °C. Blank controls consisted of identical solutions without IF-WS_2_.

The size and zeta potential were analyzed using ZetaView Software (version 8.05.16 SP3) with the following parameters: maximum area: 1000, minimum area: 10, and minimum brightness: 30. Particle concentration was calculated after subtracting blank controls. Exported Flow Cytometry Standard (FCS) files were analyzed using FCSExpress 7 (DeNovo Software, Pasadena, CA, USA), and summary graphs were created with GraphPad Prism (version 8.0).

### 2.3. PBMC Cultures

All experiments on human cells were carried out in accordance with the Declaration of Helsinki, after approval by the Ethical Committee of the Medical Faculty in Foča, University of East Sarajevo, BiH. Peripheral blood was collected from the cubital vein of healthy volunteers in citrated tubes (ThermoFisher Scientific, Waltham, MA, USA) after obtaining written informed consent. The number of donors was six, comprising both sexes, aged 20–35 years, and of different blood groups. The blood was diluted with RPMI-1640 medium (Sigma-Aldrich), layered over the Nycoprep density gradient (Nycomed, Oslo, Norway), and centrifuged. PBMCs were collected from the interphase layer.

After washing with PBS, the cells were counted using an automatic cell counter (Guava Muse Cell Analyzer, Luminex Corporation, TX, USA) and cultured in 96-well flat-bottom plates (Sarstedt, Nümbrecht, Germany) at a density of 3 × 10^5^ cells/well in 200 µL/well. The complete culture medium consisted of RPMI-1640 supplemented with 10% fetal calf serum (FCS) and antibiotics (Sigma-Aldrich): penicillin (100 U/mL), streptomycin (0.1 mg/mL), and gentamicin (0.08 mg/mL).

The cells were treated with increasing concentrations of IF-WS_2_ nanoparticles (12.5–200 µg/mL) and incubated at 37 °C in a humidified incubator with 5% CO_2_ for 24, 48, 72, or 96 h, depending on the assay. Before use, 10× concentrated IF-WS_2_ solutions were sonicated thoroughly for 10 min in an ultrasonic bath (ThermoFisher Scientific). Cultures without IF-WS_2_ served as controls. All experiments were performed in triplicates.

### 2.4. Internalization of IF-WS_2_ Nanoparticles

Cells (5 × 10^5^/well) were seeded in 24-well plates (Sarstedt) using the complete culture medium and incubated for 24 or 48 h with varying concentrations of IF-WS_2_ as described above. After incubation, the cells were collected and washed three times at low-speed centrifugation (800 rpm, 10 min) to remove unbound nanoparticles.

Following cell counting, cytospins were prepared for each sample using a cytocentrifuge (Shandon Cytospin Type 4) (ThermoFisher Scientific), and fixed with methanol (Sigma-Aldrich). After fixation and drying, cytospins were stained with May–Grünwald Giemsa (Merck, Kenilworth, NJ, USA), mounted with Canada balsam (Merck), and analyzed under a light microscope (Nikon Eclipse 5i equipped with a Nikon DXM1200C Camera, Tokyo, Japan).

Internalization of IF-WS_2_ nanoparticles (observed as black intracellular granules) was evaluated using a semiquantitative phagocytic index (PhI): PhI: 0: No stained granules; PhI: 1: Low number of small stained granules; PhI: 2: Moderate number of small and large stained granules; PhI: 3: High number of mostly large stained granules; PhI: 4: Very strong positivity, cells completely filled with dense granules. Details are provided in Section 3. The mean PhI was calculated by dividing the total internalization score by the total number of analyzed cells.

### 2.5. Viability, Apoptosis, and Necrosis Assays

PBMCs (3 × 10^5^) were cultured in 96-well plates (in triplicates) for 24 h, either untreated (control) or treated with varying concentrations of IF-WS_2_. After incubation, cells were harvested by pipetting, followed by additional PBS washing to detach adherent macrophages. The cells were pelleted by centrifugation, counted using an automatic cell counter (Guava Muse Cell Analyzer), and stained with 1% trypan blue (Merck) for viability assessment under a light microscope.

The percentage of non-viable (blue-stained) cells was calculated based on a count of 500 cells per sample. Relative viability in experimental samples was expressed as a percentage compared to control cultures (set at 100%).

For apoptosis and necrosis assays, cells were incubated in a calcium-binding buffer (R&D Systems, Abingdon, UK), and stained with Annexin V-Fluorescein isothiocyanate (FITC) and propidium iodide (PI) using the Annexin V-FITC/PI staining kit (R&D Systems), following the manufacturer’s instructions. Fluorescent cells were analyzed by flow cytometry (LSR II, Becton Dickinson, Franklin Lakes, NJ, USA).

### 2.6. Proliferation Assay

PBMCs were prestained with CellTrace Far Red dye (Invitrogen, Waltham, MA, USA) following the manufacturer’s instructions. Stained PBMCs were cultured in 96-round bottom plates at a density of 3 × 10^5^ cells/well in 200 µL of complete RPMI 1640 medium, either untreated (control) or treated with varying concentrations of IF-WS_2_. Phytohemagglutinin (PHA) (Sigma-Aldrich) at a final concentration of 20 µg/mL was used as a mitogen to stimulate cell proliferation. The cultures were incubated at 37 °C with 5% CO_2_ for 96 h.

After incubation, the cells were collected by vigorous pipetting, washed, and stained with PI at a concentration of 50 µg/mL (Sigma-Aldrich). Cell proliferation was assessed using a BD LSR II flow cytometer (BD Biosciences, San Jose, CA, USA) by analyzing the dilution of CellTrace Far Red dye, excluding doublets and PI^+^ dead cells.

T cells were isolated from PBMCs using a Pan T Cell Isolation Kit (Miltenyi Biotec, Bergisch Gladbach, Germany) based on negative immunomagnetic selection. The purity of the T cell population, determined by anti-CD3 antibody staining, exceeded 95%. Isolated T cells were activated with CD3/CD28-antibody-coated microbeads (Gibco, Thermo Fisher Scientific, Dreieich, Germany) and recombinant human IL-2 (10 ng/mL, PeproTech, Thermo Fisher Scientific). T cells were cultured under the same conditions as PHA-stimulated PBMCs for 96 h.

Proliferation analysis included calculations of the proliferation index (PI), which represents the total number of divisions divided by the number of cells that underwent division, and the division index (DI), which represents the average number of cell divisions per cell in the original population. The data were analyzed using FCS Express™ 7.20.0023 software, and the percentage of proliferating cells was also calculated. Results are presented as proliferation indices or relative values, normalized to 100% proliferation in the control group.

### 2.7. Cytokine Measurement

The concentrations of TNF-α, IFN-γ, IL-2, IL-4, IL-5, IL-6, IL-9, IL-10, IL-13, IL-17A, IL-17F, and IL-22 in the culture supernatants of PHA-stimulated PBMCs or CD3/CD28-stimulated T cells were simultaneously measured using the Flow Cytomix Microbeads Assay (BioLegend’s LEGENDplex™, San Diego, CA, USA), according to the manufacturer’s instructions. Supernatants from PHA-stimulated PBMC cultures and CD3/CD28/IL-2-stimulated T cell cultures were collected after three and four days, respectively. Cytokine concentrations were analyzed on the BD LSR II flow cytometer (BD Biosciences). A standard curve, including both positive and negative controls, was used to determine cytokine concentrations, with data analyzed using LEGENDplex™ software (2024-06-15).

The LEGEND MAX pre-coated ELISA kits for human IL-1β, IL-6, IL-8, and MCP-1 were obtained from BioLegend (Basel, Switzerland), and the Quantikine ELISA kit for GRO-α was purchased from R&D Systems (Minneapolis, MN, USA). Cytokine concentrations in the culture supernatants were determined following the manufacturer’s instructions using 5-parameter nonlinear fit curves (GraphPad Prism 8). All cytokine measurements were performed in duplicates, and the mean values were calculated. Final cytokine concentrations were standardized to the same number of cells (3 × 10^5^). The addition of WS_2_ nanoparticles to the supernatants of control PHA-stimulated PBMC cultures did not interfere with cytokine detection.

### 2.8. Statistics

To analyze differences between control and experimental groups, a repeated-measures one-way or two-way analysis of variance (RM-ANOVA), followed by Tukey’s multiple comparison test, was performed using GraphPad Prism 8 (GraphPad Software, La Jolla, CA, USA). Data are presented as means ± standard deviation (SD) from the indicated number of independent experiments, with a 95% confidence interval (*p* < 0.05) considered a significant difference between the tested groups. Where indicated, statistical significance is labeled using Compact Display Letters.

## 3. Results

### 3.1. Characterization of IF-WS_2_ Nanoparticles

IF-WS_2_ nanoparticles were initially characterized using transmission electron microscopy (TEM) and scanning electron microscopy (SEM). The results, previously published [16], are presented in Supplementary Figure S1. It can be observed that IF-WS_2_ nanoparticles are relatively symmetrical structures, composed of approximately 40 concentric layers, with diameters ranging between 40 and 70 nm. SEM analysis showed partial agglomeration, which can be attributed to their relatively high concentration (3 wt%).

**Figure 1:**
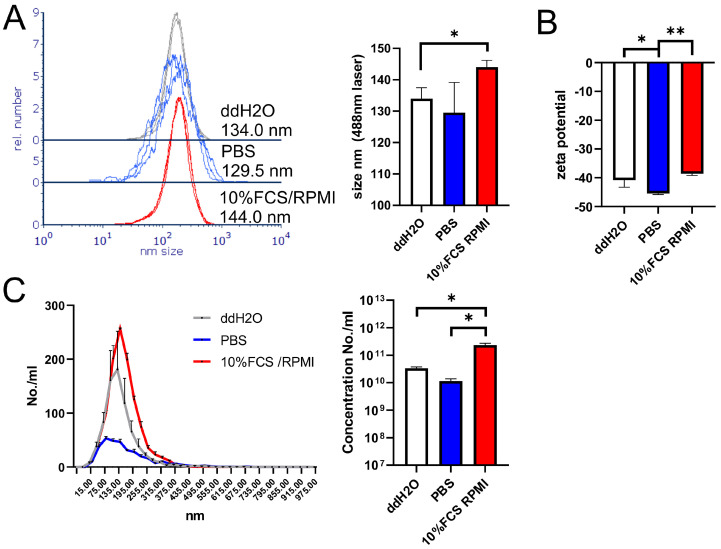
NTA measurements of IF-WS_2_ size distribution and Z-potential. (**A**) Size distribution profiles from three independent measurements are shown on a normalized *Y*-axis, with the summarized graph presented beside. (**B**) Z-potential, as measured by NTA, is shown as mean mV ± SD (*n* = 3). (**C**) Concentration distribution according to IF-WS_2_ size is shown, with each point displayed as mean ± SD (*n* = 3), and the summarized graph beside shows the mean concentration (number/mL) ± SD. * *p* < 0.05, ** *p* < 0.01 as indicated (one-way ANOVA).

Other physicochemical properties, provided by the supplier, including the range of IF-WS_2_ sizes (40–300 nm), are listed in Supplementary Table S1. NTA measurements suggested that the mean size of IF-WS_2_ in ddH_2_O is approximately 134 nm (Figure 1). IF-WS_2_ exhibited a similar size in PBS and an increased z-potential. In contrast, the size of the nanoparticles in RPMI/FCS was 10 nm larger compared to ddH_2_O, and a reduction in z-potential was observed, indicating the formation of a corona in the cell culture medium. Additionally, the concentration of IF-WS_2_ in RPMI/FCS was much higher than in water or PBS, suggesting increased stability of the IF-WS_2_ dispersion in the cell culture medium.

### 3.2. Cytotoxicity of IF-WS_2_

**Figure 2:**
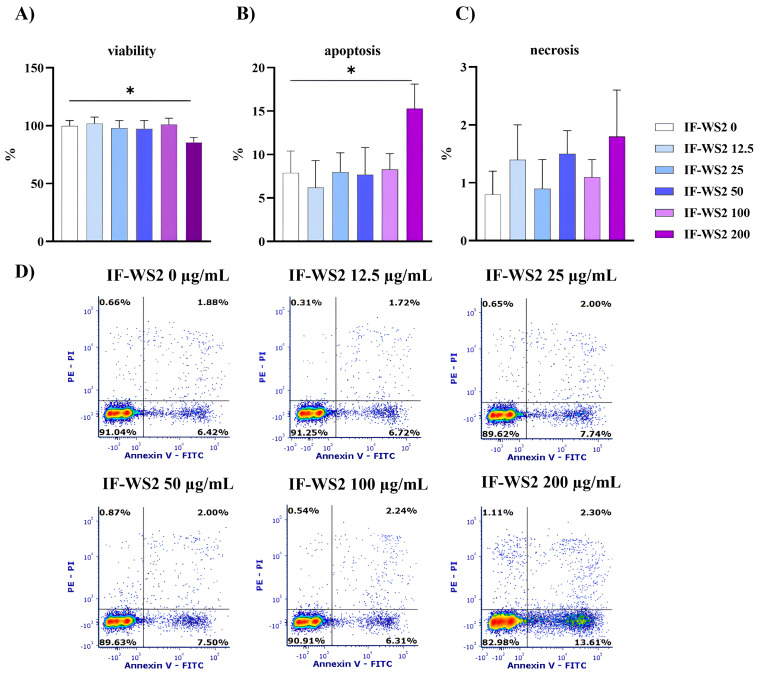
The effect of different concentrations of IF-WS_2_ nanoparticles on viability (**A**), apoptosis (**B**,**D**), and necrosis (**C**,**D**) in PBMC cultures. Values are presented as mean percentages (%) ± SD (*n* = 6) or as flow cytometric data of one representative experiment (**D**). * *p* < 0.05 compared to the control (one-way ANOVA).

The first part of the research on the biological effects of IF-WS_2_ involved investigating their impact on PBMC viability in culture. Cells were incubated with five different concentrations of IF-WS_2_ (12.5, 25, 50, 100, and 200 µg/mL) for 24 h. Control cells were cultured without IF-WS_2_ nanoparticles. Since the conventional MTT assay interfered with the color reaction, this test was not used to assess cytotoxicity. Therefore, cell viability was assessed based on the number of viable cells in cultures and calculated as a percentage relative to the control, which was taken as 100%. The results are shown in Figure 2A. It was observed that only the highest concentration slightly reduced PBMC viability by 13.6 ± 3.4% (*p* < 0.05).

The possible cytotoxic effect of IF-WS_2_ on PBMCs was further studied using an apoptosis/necrosis assay. The results confirmed the findings of the viability studies and showed the absence of cytotoxicity up to 100 µg/mL. The highest concentration (200 µg/mL) showed a slight increase in the percentage of apoptotic cells (13.3 ± 2.8%) compared to the control (7.3 ± 2.5%) (*p* < 0.05) (Figure 2B), with no significant effect on cell necrosis (Figure 2C). Flow cytometric data from one representative experiment (Figure 2D) showed a predominant increase in early apoptotic (Annexin-V-FITC+/PI-) cells in the presence of the highest concentration of IF-WS_2_.

### 3.3. Internalization of IF-WS_2_ Nanoparticles by PBMCs

**Figure 3:**
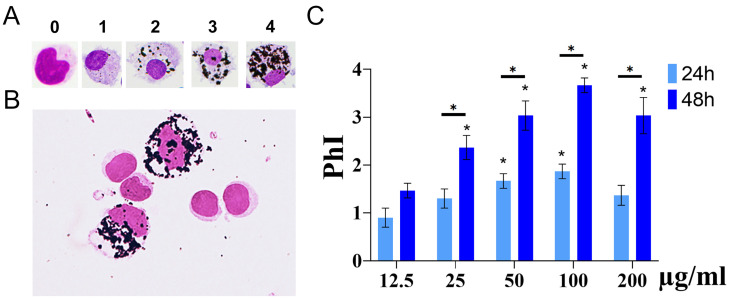
Internalization of IF-WS_2_ nanoparticles in PBMC cultures. (**A**) Heterogeneity of IF-WS_2_ uptake by macrophages after 48 h, where phagocytic indexes (PhI) are indicated above the cells. (**B**) Internalization of IF-WS_2_ by macrophages, but not lymphocytes, analyzed after 48 h using cytospins. (**C**) The dose- and time-dependent effect of IF-WS_2_ internalization by macrophages is shown. Values are expressed as mean ± SD of PhI (*n* = 3), determined by calculating PhI for 500 cells on each cytospin. * *p* < 0.05 compared to the lowest concentration (12.5 µg/mL) or as indicated by bars and determined by two-way ANOVA followed by Tukey’s multiple comparison test.

**Figure 4:**
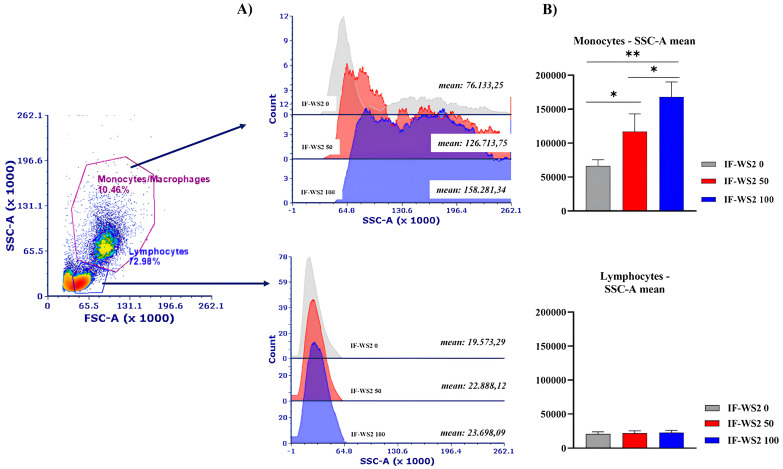
Internalization of IF-WS_2_ by PBMCs in culture. PBMCs were cultured with IF-WS_2_ for 24 h, followed by analysis by flow cytometry. (**A**) Gating strategy showing the population of monocytes/macrophages (above) and lymphocytes (below). (**B**) Mean values of size scatter for each population (monocytes/macrophages above, lymphocytes below) are presented. Data are shown as mean ± SD (*n* = 3), with representative histograms included. * *p* < 0.05; ** *p* < 0.01 as indicated on the horizontal bars (one-way ANOVA).

PBMCs were cultured with different concentrations of IF-WS_2_. After incubation, the cells were collected and prepared for morphological and flow cytometric analysis. Morphological analysis was performed on cytospins prepared from PBMCs. To study the extent of internalization depending on the concentrations of IF-WS_2_ and incubation time, a semiquantitative analysis was performed, as presented in Figure 3A. Figure 3B shows that internalized IF-WS_2_ nanoparticles were detected in monocytes/macrophages, but not in lymphocytes. An increase in internalization was observed after 24 h in the culture at concentrations up to 100 µg/mL, with significant differences (*p* < 0.05) observed when comparing PhI (50 µg/mL) and PhI (100 µg/mL) to the lowest concentration (12.5 µg/mL). A more pronounced increase in internalization was observed after 48 h, with statistically significant differences between all concentrations compared to the lowest one (*p* < 0.05). Additionally, all PhI values were statistically significantly higher (*p* < 0.05) compared to the corresponding PhI after 24 h, except for the lowest concentration (Figure 3C). Interestingly, at the highest concentration of IF-WS_2_ nanoparticles (200 µg/mL), the degree of internalization was slightly lower than at 100 µg/mL. Flow cytometry data confirmed that monocytes/macrophages internalize IF-WS_2_, as indicated by a concentration-dependent increase in scatter, in contrast to lymphocytes, where such changes were not observed (Figure 4).

### 3.4. The Effect of IF-WS_2_ on PBMC Proliferation

**Figure 5:**
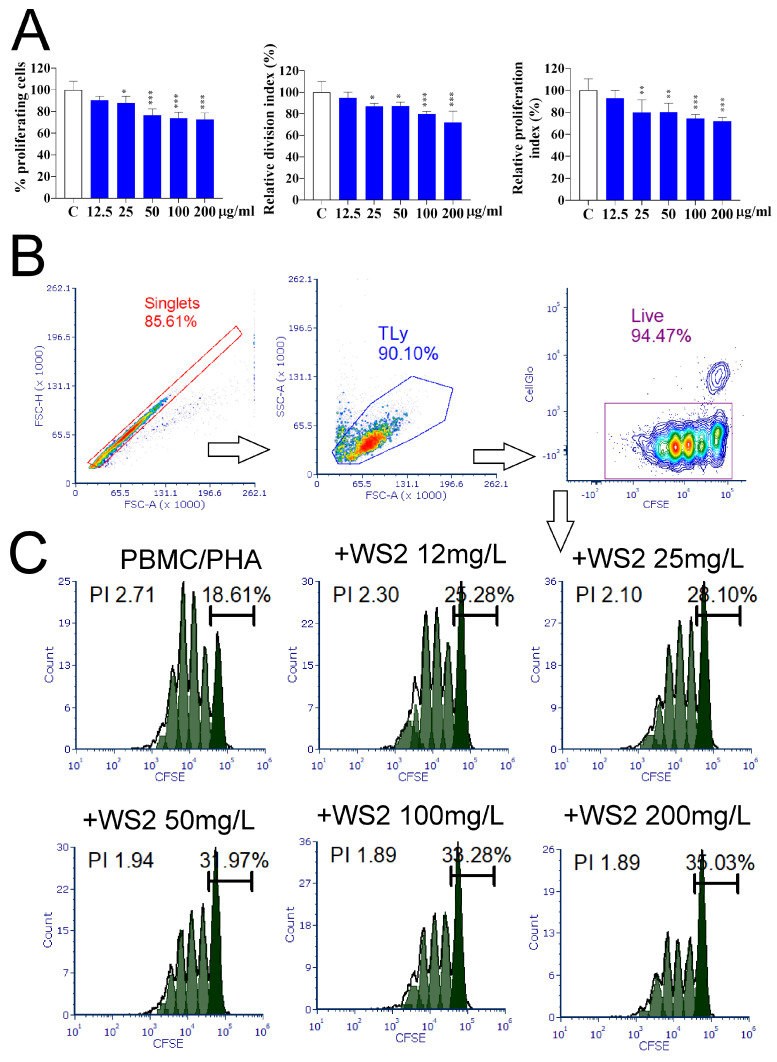
The effect of IF-WS_2_ nanoparticles on the proliferation of PHA-stimulated PBMCs. (**A**) Cell proliferation parameters are presented as relative values (%) ± SD (*n* = 3), with control cultures used as 100%. * *p* < 0.05; ** *p* < 0.01; *** *p* < 0.005 compared to the control (one-way ANOVA). (**B**) Gating strategy. (**C**) Representative histograms from one experiment. The percentages above the bars represent the proportion of non-dividing cells. PI—proliferating index.

PBMCs prestained with CellTrace Far Red were stimulated with PHA for 96 h. Experimental cultures were treated with five different concentrations of IF-WS_2_ (12.5, 25, 50, 100, and 200 µg/mL). Afterward, cell proliferation was analyzed using a flow cytometer. Several parameters, such as PI, DI, and the percentage of proliferating cells, were determined and expressed as relative values, with 100% representing the parameters in the control (Figure 5A). It can be observed that IF-WS_2_ concentrations of 25 µg/mL and higher inhibited cell proliferation. The inhibition ranged from 26.4% to 32.6% at the highest concentration, depending on the parameter investigated (*p* < 0.005). Representative histograms from one experiment are shown in Figure 5B.

PHA is a T cell mitogen. To investigate whether IF-WS_2_ directly inhibited T cell proliferation, purified T cells were stimulated with microbeads coated with CD3/CD28 antibodies and IL-2. The results presented in Supplementary Table S2 show that neither concentration of IF-WS_2_ inhibited T cell proliferation.

### 3.5. The Effect of IF-WS_2_ Nanoparticles on the Production of Pro-Inflammatory Cytokines and Chemokines in PHA-Stimulated PBMC Cultures

**Figure 6:**
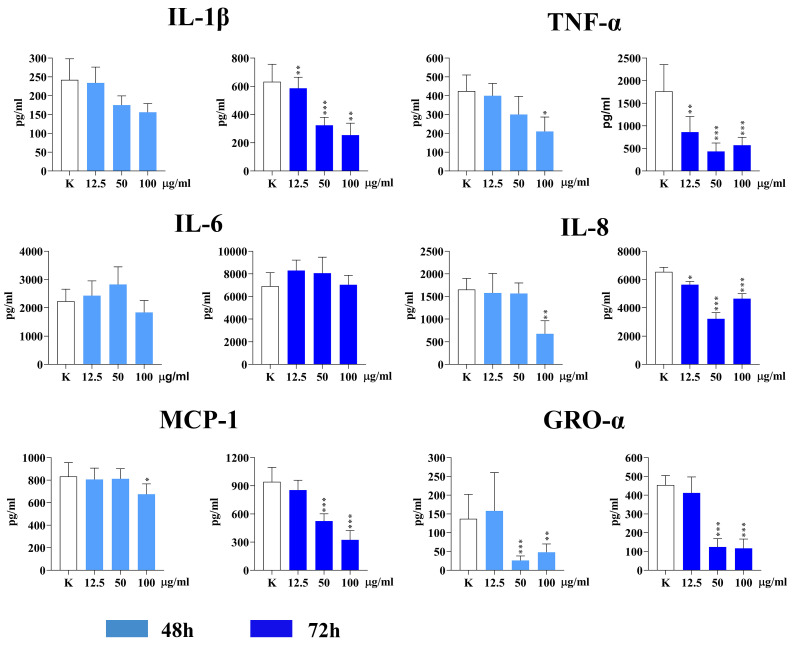
The effect of IF-WS_2_ nanoparticles on the production of pro-inflammatory cytokines and chemokines in PHA-stimulated PBMC cultures. Values are presented as mean ± SD (*n* = 3). * *p* < 0.05; ** *p* < 0.01; *** *p* < 0.005 compared to the control (one-way ANOVA).

The concentrations of three pro-inflammatory cytokines (IL-1β, IL-6, and TNF-α) and three chemokines (IL-8, MCP-1, and GRO-α) were measured in the supernatants of PBMC cultures stimulated with PHA after 48 and 72 h. Double-increasing concentrations of IF-WS_2_ nanoparticles, ranging from 12.5 µg/mL to 100 µg/mL, were used. The results are presented in Figure 6.

No significant changes in the levels of IL-1β and IL-6 were observed after 48 h. However, the levels of TNF-α, IL-8, and MCP-1 were significantly lower at the concentration of 100 µg/mL (*p* < 0.05) compared to control cultures. Additionally, GRO-α levels were most significantly decreased at 50 and 100 µg/mL (*p* < 0.005 and *p* < 0.01, respectively).

After 72 h, the levels of all cytokines (except for IL-6) changed significantly in the presence of IF-WS_2_. All three concentrations of nanoparticles significantly decreased the levels of IL-1β, TNF-α, and IL-8 (*p* < 0.05, *p* < 0.01, or *p* < 0.005). Furthermore, MCP-1 and GRO-α levels were significantly decreased at 50 and 100 µg/mL of IF-WS_2_ (*p* < 0.005).

### 3.6. The Effect of IF-WS_2_ Nanoparticles on the Production of Th1, Th2, and Th9 Cytokines in PHA-Stimulated PBMC Cultures

**Figure 7:**
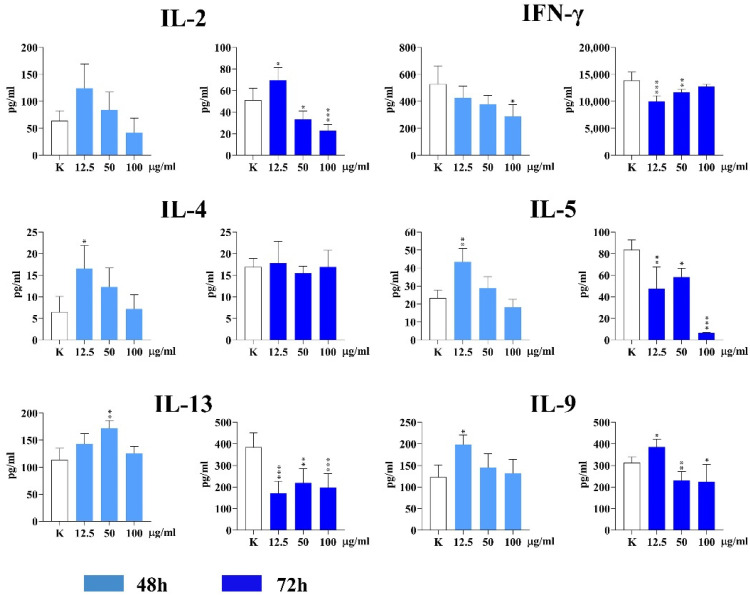
The effect of IF-WS_2_ nanoparticles on the production of Th1, Th2, and Th9 cytokines in PHA-stimulated PBMC cultures. Values are given as mean ± SD (*n* = 3). * *p* < 0.05; ** *p* < 0.01; *** *p* < 0.005 compared to the control (one-way ANOVA).

The level of IL-2 (a common T cell proliferating cytokine) did not significantly change in the presence of IF-WS_2_ after 48 h of PBMC culture. However, the lowest IF-WS_2_ concentration (12.5 µg/mL) stimulated IL-2 production (*p* < 0.05), while the other two concentrations (50 and 100 µg/mL) significantly inhibited IL-2 production (*p* < 0.05 and *p* < 0.005, respectively). The results are presented in Figure 7.

The concentrations of IF-WS_2_ (12.5 and 50 µg/mL) significantly decreased the levels of IFN-γ (a key Th1 cytokine) after 72 h (*p* < 0.05 and *p* < 0.01, respectively). Interestingly, the highest concentration of the nanoparticles decreased the production of IFN-γ after 48 h (*p* < 0.05), but not after 72 h.

The production of three Th2 cytokines (IL-4, IL-5, and IL-13) was measured in the culture supernatants. The levels of IL-4 (*p* < 0.05) and IL-5 (*p* < 0.01) were significantly higher in the presence of the lowest concentration of IF-WS_2_ after 48 h, whereas an increased level of IL-13 (*p* < 0.01) was detected at 50 µg/mL at the same time. After prolonged cell culture (72 h), the stimulatory effect of IF-WS_2_ was not observed. In contrast, all three concentrations of nanoparticles inhibited the production of IL-5 and IL-13 (*p* < 0.01 or *p* < 0.005). The production of IL-4 was not significantly modulated.

The lowest concentration of IF-WS_2_ significantly stimulated IL-9 production (a key Th9 subset cytokine) (*p* < 0.05) at both 48 and 72 h. After 72 h, the other two concentrations of IF-WS_2_ significantly inhibited IL-9 production (*p* < 0.01 and *p* < 0.05, respectively).

### 3.7. The Effect of IF-WS_2_ Nanoparticles on the Production of Th17, Th21, and Treg Cytokines in PHA-Stimulated PBMC Cultures

**Figure 8:**
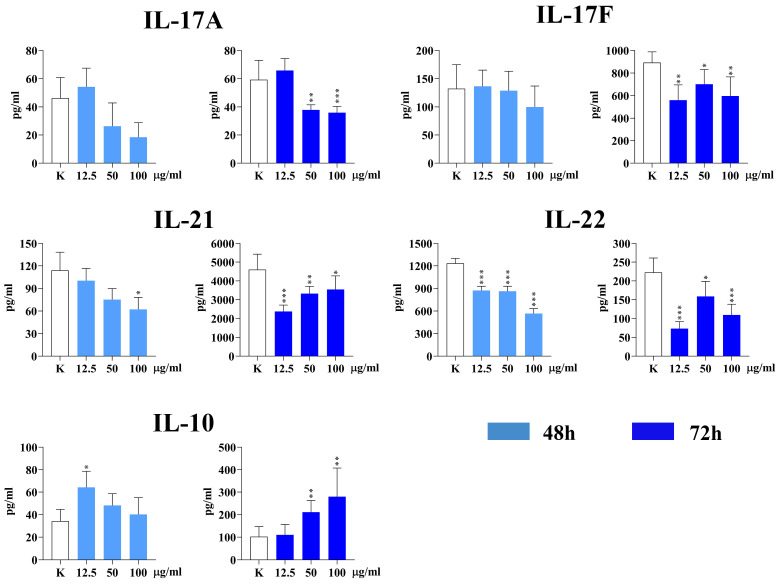
The effect of IF-WS_2_ nanoparticles on the production of Th17, Th21, and Treg cytokines in PHA-stimulated PBMC cultures. Values are presented as mean ± SD (*n* = 3). * *p* < 0.05; ** *p* < 0.01; *** *p* < 0.005 compared to the control (one-way ANOVA).

The following Th17 cell subset cytokines (IL-17A, IL-17F, and IL-22), Th21 cytokine (IL-21), and Treg cytokine (IL-10) were analyzed in the culture supernatants of PHA-stimulated PBMC cultures. The results are presented in Figure 8.

Regarding Th17 cytokines, only IL-22 was significantly decreased after 48 h in the presence of all concentrations of IF-WS_2_ (*p* < 0.005). However, the levels of all three Th17 cytokines were decreased after 72 h.

All concentrations of IF-WS_2_ inhibited the production of IL-21 after 72 h (*p* < 0.005, *p* < 0.01, and *p* < 0.05, respectively), as did the highest concentration after 48 h (*p* < 0.05). The lowest concentration of IF-WS_2_ stimulated the production of IL-10 (*p* < 0.05) after 48 h, as did the other two concentrations after 72 h (*p* < 0.01).

### 3.8. The Effect of IF-WS_2_ on the Production of T Helper Cytokines in CD3/CD28-Stimulated T Cell Cultures

When purified T cells were stimulated with anti-CD3/CD28 microbeads, no significant changes in the production of any of the investigated T cell cytokines were detected in the presence of IF-WS_2_ nanoparticles compared to the control. The levels of IL-2 were not determined, as the cytokine was already present in the culture system (Supplementary Table S3).

## 4. Discussion

This study aimed to investigate the effect of different concentrations of IF-WS_2_ on human PBMCs in culture, serving as an initial screening for the potential modulatory effects of these nanoparticles on immune responses. PHA, a well-known mitogen for T lymphocytes, was used to stimulate the proliferation and activation of PBMCs, for which the presence of accessory cells in the culture (monocytes and dendritic cells) is necessary for its effect [17,18].

IF-WS_2_ nanoparticles used in this study have been previously well characterized [8,16]. Since we dispersed them in a culture medium containing serum proteins, it was necessary to characterize their physicochemical properties under such culture conditions. In this context, we used NTA as an alternative and complementary method to dynamic light scattering (DLS) [19], which sizes particles from 30 to 1000 nm. The increase in the size of IF-WS_2_ in a culture medium supplemented with 10% FCS, along with a reduction of z-potential and increased stability, suggests that the nanoparticles are surrounded by a protein corona, most likely composed primarily of albumin [20]. However, neither DLS or NTA are optimal for detecting larger aggregates [19], which are visible by inverted microscopic analysis but difficult to quantify accurately.

Before conducting immunological tests, it was necessary to assess the cytotoxicity of IF-WS_2_. The most commonly used cytotoxicity screening (MTT test) could not be performed because these nanoparticles interfered with the colorimetric reaction. Therefore, the potential cytotoxic effect was assessed based on viability and apoptosis/necrosis assays. The results showed minimal cytotoxicity, observed only at the highest concentration of IF-WS_2_, consistent with earlier findings of the biocompatibility of these nanoparticles in rat salivary gland cells [8], A545 [9], kidney cell lines [10], or human monocyte-derived macrophages [21]. This contrasts with the study by Teo et al. (2014), which demonstrated varying cytotoxicity of lithiated WS_2_, MoS_2_, and WSe_2_, which are chemically and electronically different from their pristine forms [22].

Although non-cytotoxic, IF-WS_2_ inhibited cellular proliferation. As expected, the inhibitory effect was relatively small and increased with higher concentrations of IF-WS_2_ (up to a maximum of about 30%), but it was not clearly dose-dependent. Since IF-WS_2_ did not affect the proliferation of purified T cells stimulated with anti-CD3/CD28 microbeads, it can be assumed that the effect is indirect and dependent on accessory cells in the culture. This is supported by the results of nanoparticle internalization analysis, which showed that IF-WS_2_ were internalized (phagocytosed) by monocytes/macrophages but not by lymphocytes. Our results differ from those published on salivary gland cells, which showed unimpaired cell proliferation using IF-WS_2_ of the same company used in this study [8]. In contrast, another study on tungstate (Na_2_WO_4_) showed inhibited proliferation of PB leukocytes due to a prominent cytotoxic effect [23]. The variability in these results can be explained by differences in the cell types used and the different forms and compositions of tungsten materials.

Macrophages are the most potent phagocytic cells and are the first cells that encounter foreign bodies, including nanoparticles [24]. Metallic nanoparticles are particularly significant due to their long persistence and difficulty in elimination once ingested. Given that the internalization of nanoparticles depends on particle geometry, surface charge, functionalization, serum protein opsonization, and other surface parameters, as well as culture conditions [24,25], it can be postulated that macrophages internalize IF-WS_2_ via phagocytosis. This is the primary uptake mechanism for large nanoparticles, including those that are aggregated (reversible process) or agglomerated (irreversible process). Macrophages are differentiated from monocytes in PBMC cultures. The nanoparticles ingested via phagocytosis fuse with lysosomes to form phagosomes, and our microscopic analysis strongly suggests that large, dark intracytoplasmic structures are phagolysosomes. Other mechanisms of nanoparticle uptake, such as macropinocytosis (large-volume uptake of nanoparticles larger than 500 nm), clathrin-dependent endocytosis (which operates for nanoparticles of 20–500 nm), and caveolin-mediated endocytosis (responsible for the uptake of 20–100-nm particles) [26,27], could also be relevant. This hypothesis is supported by the findings of Peng et al. using a similar cell culture model with human monocyte-derived macrophages treated with MoS_2_ or WS_2_ 2D exfoliated nanosheets, where cytochalasin D partially reduced nanoparticle uptake [21]. It is known that cytochalasin D inhibits actin-dependent uptake mechanisms, such as phagocytosis or macropinocytosis [28]. We also used flow cytometry to demonstrate the uptake of WS_2_ nanoparticles by macrophages. Although this method could not clearly distinguish between internalized nanoparticles and those attached to the membrane, it correlates with morphological features associated with internalization. Furthermore, it provides additional confirmation when analyzing lymphocytes, which neither bind to nor internalize WS_2_ nanoparticles.

Once internalized by macrophages, nanoparticles may affect their cellular physiology in various ways. The most commonly studied function of macrophages relates to the production of biomolecules associated with the inflammatory response. This is why we examined the production of pro-inflammatory cytokines (IL-1β, TNF-α, IL-6) and chemokines (IL-8, GRO-α, MCP-1). TNF-α and IL-1β are typical examples of cytokines produced primarily by innate immune cells, especially macrophages. They are important for both acute and chronic inflammation, acting on different components of the inflammatory cascade. IL-8 and GRO-α have a similar pro-inflammatory role, primarily mobilizing granulocytes. On the other hand, MCP-1 is mainly involved in recruiting monocytes to the inflammatory site [29]. We found that IF-WS_2_ exerted significant suppression of all investigated cytokines/chemokines, except for IL-6, which was not significantly affected. The effect was more pronounced after prolonged PBMC culture and PHA stimulation, which is consistent with the increased internalization of IF-WS_2_ over time. The unchanged production of IL-6 could be due to its dual pro- and anti-inflammatory roles [30].

Our results are comparable to studies on metallic nanoparticles. Aili et al. recently demonstrated that Au nanoparticles suppress neuroinflammation by inducing macrophage polarization toward the M2 phenotype in a microglia model, reducing pro-inflammatory (TNF-α and IL-1β) cytokine expression through inhibition of the NF-kB and MAPK signaling pathways, blocking leukocyte adhesion, and decreasing oxidative stress [31]. A similar effect was observed with Au-deposited metal oxide nanoparticles, which down-modulated TNF-α and IL-1β secretion by a murine macrophage cell line [32]. Peng et al. [21] showed that 2D MoS_2_ and WS_2_ materials were non-cytotoxic to primary human macrophages and did not provoke significant pro-inflammatory effects. Tungstate (mainly polyoxotungstate-1, 3Na_2_WO_4_·9WO_3_·H_2_O) up to 100 µM did not stimulate ROS production, cause DNA damage, or influence the production of IL-6, IL-8, and TNF-α in RAW 264.7 macrophages [33]. In contrast, many nanoparticles can stimulate pro-inflammatory responses in macrophages, manifesting as increased ROS production, secretion of pro-inflammatory cytokines, and upregulation of activation markers [34].

The most important findings in our study relate to T cell cytokine production. We showed that IF-WS_2_ significantly modulated Th responses, which depend on antigen-presenting cells in PHA-stimulated PBMC cultures. The levels of pro-inflammatory Th1 (IFN-γ) and Th17 (IL-17A, IL-17F, and IL-22) cytokines, as well as Th21 (IL-21), were significantly decreased, while the levels of anti-inflammatory Th2 cytokines (IL-4, IL-5, IL-13), Th9 (IL-9), and the key immunoregulatory cytokine IL-10 were significantly increased.

Although Th1 cells generally have a pro-inflammatory role and contribute to certain autoimmune diseases, they are also responsible for immunity against intracellular microorganisms and the stimulation of anti-viral and anti-tumor cytotoxic CD8+ T cell responses [35]. The role of Th17 cells in immune responses is complex. Th17 cells stimulate immunity against extracellular bacteria and fungi, support acute inflammation, and are involved in the pathogenesis of several autoimmune diseases and allergies [36]. Their role in cancer immunity is controversial. IL-21 is a pleiotropic cytokine primarily produced by the Th21 subset. It stimulates cytotoxicity in CD8+ T cells and NK cells and participates in forming immunological memory through interactions between T follicular helper cells and B cells in germinal centers of lymphoid organs. IL-21 is also implicated in many systemic autoimmune diseases [37]. According to our results, the inhibited production of Th1, Th17, and Th21 cytokines represents an additional anti-inflammatory immune response induced by IF-WS_2_ nanoparticles.

Interestingly, the Th2 response was increased in the early phase of activation (48 h) at lower concentrations of IF-WS_2_, while the production of IL-5 and IL-13 decreased after 72 h at all concentrations used. In contrast, IL-4 production remained unchanged. These results suggest that while Th2 cells promote anti-helminthic responses, asthma, and other allergies, as well as some chronic infections and humoral immunity, the production of individual Th2 cytokines is regulated differently [38]. Th9 cells, through IL-9 production, play a role in various immune-related diseases, including tumors, inflammatory diseases, parasite infections, and asthma [39]. The differentiation of Th9 cells is regulated by TGF-β and IL-4, and the upregulated production of IL-9 by IF-WS_2_ follows a similar pattern to the Th2 response. However, the kinetics of IL-9 and Th2 cytokines differed, as IL-9 production remained elevated after 72 h.

The increased production of IL-10 aligns with the enhanced anti-inflammatory and immunoregulatory response in IF-WS_2_-treated PBMCs. IL-10 is produced by various cell types, including macrophages and Treg cells. In addition to its inhibitory effect on pro-inflammatory cytokine production by innate immune cells, IL-10 also suppresses Th1, Th17, and Th9 responses [40], all of which were observed in our study. Th2 cytokines and IL-10 may also promote the differentiation of M2 macrophages, which are more effective in engulfing nanoparticles than M1 macrophages [33]. These M2 macrophages, through increased uptake of IF-WS_2_, may further enhance the anti-inflammatory microenvironment in our culture system.

A key question in explaining our results is the mechanism by which IF-WS_2_ strongly modulates the T cell response via accessory cells (primarily macrophages), as no effect was observed when purified T cells were used. Several possibilities exist. First, upon engulfing IF-WS_2_, macrophages could transform into the M2 phenotype, as suggested by studies using co-culture systems of A549 and THP-1 cells [41] or microglial cells exposed to Au nanoparticles [32]. Activated M2 macrophages express immunoregulatory molecules such as PD-1L and B7-H4 and produce several immunosuppressive factors, including arginase, indoleamine-2,3-dioxygenase (IDO), IL-10, TGF-β, and various small non-coding RNAs [42]. These functions of M2 macrophages closely resemble those of tumor-infiltrating macrophages. IL-4 secreted by M2 macrophages stimulates the maturation of naïve T cells into Th2 cells, which, in turn, secrete IL-4 and IL-13 [42]. As previously mentioned, IL-10 inhibits CD8+ T cell-dependent immune responses [30] and promotes the accumulation of Tregs [42]. Furthermore, cytokines like IL-25 (IL-17E) and IL-33 (alarmins) produced by activated macrophages can stimulate Th2 immune responses while inhibiting Th1 and Th17 responses through suppression of IL-23 and IL-12, respectively [39]. Cytokines produced by Th2 cells and M2 macrophages may inhibit NF-kB activity and, consequently, be responsible for suppressing the pro-inflammatory response in the presence of WS_2_ nanoparticles. Nevertheless, this hypothesis requires further investigation.

The macrophage/T cell interaction could be driven by the so-called bystander effect of nanoparticles, which has been demonstrated in co-cultures of lung cells and macrophages exposed to WS_2_. In these models, WS_2_ nanosheets induce M2 polarization in macrophages [41]. In our culture system, this phenomenon might translate into a macrophage-induced bystander effect on T cells.

Another potential mechanism involves exosomes containing WS_2_ nanoparticles released from macrophages. A recent study [43] showed that Au nanoparticles, after entering a keratinocyte cell line and trafficking to multivesicular bodies and lysosomes, exited the cell primarily through unconventional exocytosis. These exosomes, measuring up to 100 nm and expressing CD81, could deliver their cargo intracellularly, thereby influencing immune responses. According to known exosome-mediated cell-cell communication mechanisms, IF-WS_2_-containing exosomes may be taken up by T cells [44], potentially modulating the immune response. A recent study also demonstrated that exosomes released from M2 macrophages could promote the differentiation of naïve T cells into Treg cells [45].

The results presented here offer valuable starting points for future experiments, despite the limitations outlined in the discussion. These findings inspire new lines of inquiry, particularly regarding the specific ways in which T cells respond within a microenvironment that contains free IF-WS_2_ and macrophages with ingested IF-WS_2_. Key questions remain, such as how cytokine profiles evolve over cultivation time and what the final outcome of these processes might be. One approach to further understanding could involve analyzing exosomes released from IF-WS_2_-ingested macrophages. This is why we characterized the physicochemical properties of IF-WS_2_ and their changes in culture medium using NTA, a method commonly employed for exosome analysis.

## 5. Conclusions

This study demonstrates that pristine IF-WS_2_ nanoparticles are cytocompatible and exhibit significant anti-proliferative, anti-inflammatory, and immunomodulatory effects in a human PBMC culture model, an observation not previously published. The anti-inflammatory response of activated immune cells to IF-WS_2_, coupled with the downregulation of Th1, Th17, and Th21 responses, positions this dichalcogenide nanostructures as a promising biomaterial for the treatment of autoimmune diseases and transplantation-related reactions. However, these effects may not be desirable for applications in anti-tumor immunity and allergy. Nevertheless, by functionalizing IF-WS_2_ with appropriate immunostimulants, along with leveraging their excellent photothermal properties, these nanoparticles could become an attractive candidate for tumor immunotherapy. However, before their therapeutic application in vivo, extensive preclinical studies using experimental animals are required to validate the observed in vitro phenomena and establish their correlation with pharmacokinetic data.

## Data Availability

The data are contained within the article.

## Supplementary Materials

The following supporting information can be downloaded at: https://www.mdpi.com/article/10.3390/nano15050322/s1, Figure S1: Characterization of IF-WS_2_ nanoparticles by TEM (a) and SEM (b). Taken from: Simić et al. Composites Part B 176 (2019) 107222, with the permission of Elsevier.; Table S1: Properties of the inorganic fullerene-like tungsten disulfide nanoparticles (IF-WS_2_); Table S2: Effect of different concentrations of IF-WS_2_ nanoparticles on proliferation of purified T cells stimulated with CD3/CD28 beads and IL-2; Table S3: Effect of different concentrations of IF-WS_2_ on the production of cytokines by T cells stimulated with anti-CD3/CD28 microbeads.
