# Supplementary material for: Anti-Inflammatory and Immunomodulatory Properties of Inorganic Fullerene-Like Tungsten Disulfide Nanoparticles in the Culture of Human Peripheral Blood Mononuclear Cells
Source: Nanomaterials (Basel). 2025 Feb 20;15(5):322. doi: 10.3390/nano15050322 (PMC11901739; doi:10.3390/nano15050322)
Supplement: Supplementary file 1 [file nanomaterials-15-00322-s001.zip › nanomaterials-3399781-supplementary.pdf]

Supplementary materials

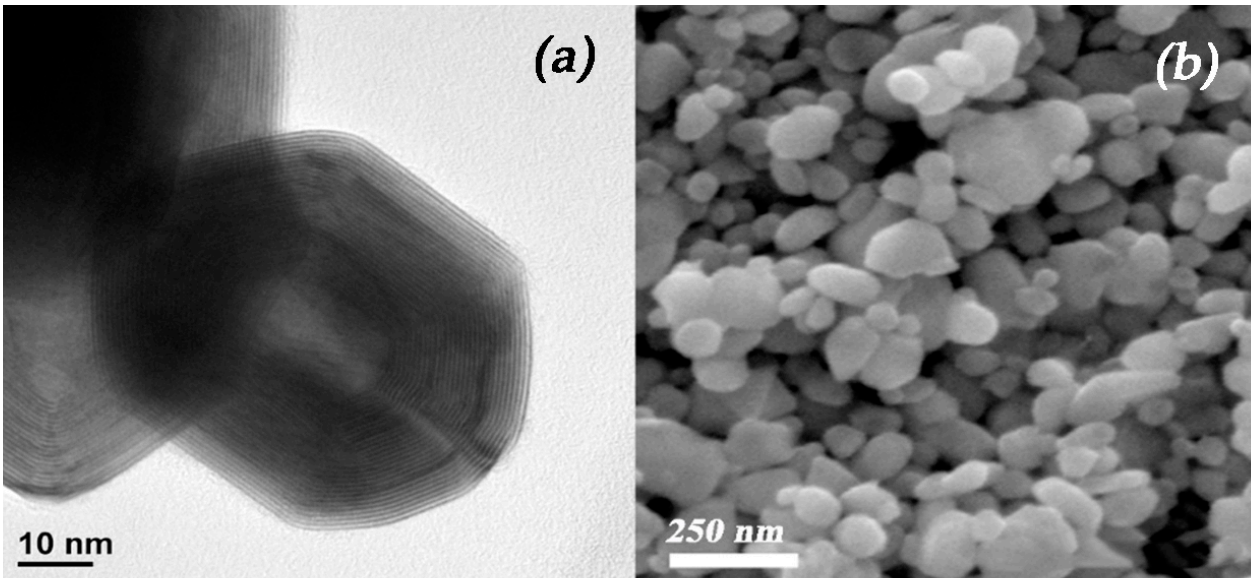

Supplementary Figure S1. Characterization of IF-WS_2 nanoparticles by TEM (a) and SEM (b). Taken from: Simić et al. Composites Part B 176 (2019) 107222, with the permission of Elsevier.

Supplementary Table S1. Properties of the inorganic fullerene-like tungsten disulfide nanoparticles (IF-WS_2)

Property	IF-WS_2
Purity	> 99%
Particle density, at 25 °C	7.5 g/cm3
Apparent (bulk) density	0.7-1.1 g/cm3
Typical particle size/ outer diameter	40-300 nm
Decomposition temperature	1250°C
Oxidation stability, air	>350°C
Oxidation stability, inert	>1000°C
Molecular weight	247.98 g/mol

The data were obtained from the producer (NanoLub™ ApNano, Israel)

Supplementary Table S2. Effect of different concentrations of IF-WS_2 nanoparticles on proliferation of purified T cells stimulated with CD3/CD28 beads and IL-2

Parameters	IF-WS_2 (µg/mL)				
	12.5	25	50	100	200
Proliferation index	4.2 ± 0.3	4.4 ± 0.3	4.1 ± 0.2	3.9 ± 0.4	3.9 ± 0.2
Proliferating cells (%)	100 ± 5.5	103 ± 5.0	105 ± 4.8	100 ± 6.1	98 ± 4.4
Division index	3.1 ± 0.2	3.3 ± 0.3	3.0 ± 0.3	3.0 ± 0.4	2.9 ± 0.3

Values are given as mean ± SD (n = 3)

Supplementary Table S3. Effect of different concentrations of IF-WS_2 on the production of cytokines by T cells stimulated with anti-CD3/CD28 microbeads

IF-WS_2 (µg/mL)	Cytokines (pg/mL)								
	IFN-γ	IL-4	IL-5	IL-13	IL-17A	IL-9	IL-22	IL-21	IL-10
0	2543 ± 443	86.4 ± 23.2	96.4 ± 25.2	116.4 ± 30.3	122.2 ± 33.8	142.2 ± 36.0	162.5 ± 28.8	307.1 ± 24.5	66.0 ± 18.8
12.5	2245 ± 322	76.2 ± 18.2	106.0 ± 20.2	110.8 ± 20.5	108.2 ± 27.8	128.8 ± 31.7	158.4 ± 32.0	318.4 ± 38.3	92.2 ± 25.0
25	2454 ± 286	80.4 ± 27.4	112.4 ± 33.0	132.4 ± 38.3	143.2 ± 32.4	112.2 ± 38.0	148.6 ± 30.4	288.8 ± 31.6	58.4 ± 14.2
50	2156 ± 350	69.0 ± 21.9	86.0 ± 26.2	106.4 ± 21.3	120.2 ± 35.2	134.9 ± 30.6	170.2 ± 45.2	290.1 ± 31.0	68.2 ± 11.6
100	2207 ± 198	76.4 ± 14.6	80.4 ± 21.5	96.4 ± 27.2	110.2 ± 22.0	118.5 ± 25.8	150.8 ± 33.0	286.5 ± 30.4	70.2± 13.2

Values are given as mean ± SD (n = 3). All differences were $p > 0.05$ compared to the corresponding controls
